# In Vitro Antibacterial Effects of *Amygdalus scoparia* (Zedo Gum) Hydroalcoholic Extract on Oral Streptococci and *Staphylococcus aureus* Compared With Chlorhexidine

**DOI:** 10.1155/ijod/3823885

**Published:** 2026-07-27

**Authors:** Seyedeh Zahra Miri, Arash Asfaram, Razieh Dehbanipour, Mobina Moeindoost, Fatemeh Khajehzadeh

**Affiliations:** ^1^ Faculty of Dentistry, Yasuj University of Medical Sciences, P.O. Box 7591873149, Yasuj, Iran, yums.ac.ir; ^2^ Medicinal Plants Research Center, Yasuj University of Medical Sciences, Yasuj, Iran, yums.ac.ir; ^3^ Department of Microbiology, School of Medicine, Yasuj University of Medical Sciences, P.O. Box 7591873149, Yasuj, Iran, yums.ac.ir; ^4^ Department of Oral and Maxillofacial Pathology, Faculty of Dentistry, Yasuj University of Medical Sciences, P.O. Box 7591873149, Yasuj, Iran, yums.ac.ir

**Keywords:** antibacterial agents, chlorhexidine, oral, plant extracts, Rosaceae, *S. mutans*

## Abstract

**Background and Objective:**

Due to escalating concerns regarding bacterial resistance and the adverse effects associated with chlorhexidine, natural antimicrobial agents have gained significant attention as potential alternatives. The present study aimed to evaluate the in vitro antibacterial activity of the hydroalcoholic extract of Amygdalus scoparia (*A. scoparia*) gum against primary oral streptococci and *Staphylococcus aureus*, comparing its efficacy with chlorhexidine and conventional antibiotics.

**Materials and Methods:**

The hydroalcoholic extract of *A. scoparia* gum was tested against standard and clinical isolates of *Streptococcus mutans* (*S. mutans*), *Streptococcus sobrinus* (*S. sobrinus*), *Streptococcus sanguinis* (*S. sanguinis*), *and S. aureus*. Antibacterial activity was assessed using the agar well diffusion method to determine the zone of inhibition (ZOI) and the broth microdilution method to determine the minimum inhibitory concentration (MIC) and minimum bactericidal concentration (MBC). Chlorhexidine (0.2%) and antibiotics (vancomycin and penicillin) served as positive controls. All experiments were performed in triplicate.

**Results:**

The *A. scoparia* extract demonstrated significant antibacterial activity against all tested microorganisms. Notably, the extract produced a significantly larger ZOI against clinical isolates of *S. mutans* (9.40 ± 0.97 mm) compared with chlorhexidine (8.50 ± 1.08 mm; *p* = 0.0448). Although chlorhexidine exhibited the lowest MIC and MBC values (2.00–13.33 µg/mL), the plant extract showed moderate inhibitory effects (MIC range: 10.67–250.00 mg/mL), particularly against cariogenic streptococci. Overall, clinical isolates demonstrated greater resistance than standard strains.

**Conclusion:**

The hydroalcoholic extract of *A. scoparia* gum exhibited promising antibacterial activity against key oral pathogens, especially clinical isolates of *S. mutans*, showing comparable or superior efficacy to chlorhexidine in agar diffusion assays. These findings suggest that this extract holds potential as a natural adjunct or alternative antimicrobial agent in oral healthcare products.

## 1. Introduction

Oral health is a fundamental component of holistic health, exerting a pivotal influence on systemic well‐being and quality of life. Despite considerable advancements in preventive dentistry, the global prevalence of oral diseases remains alarmingly high [1, 2]. Current estimates suggest that ~3.5 billion individuals worldwide are affected by oral diseases, particularly dental caries. Caries arise from a microbial imbalance within the complex oral microbiota, which encompasses over 700 distinct bacterial species [3, 4].

Among the diverse microorganisms implicated in oral pathologies, *Streptococcus mutans* (*S. mutans*), *Streptococcus sobrinus* (*S. sobrinus*), and *Streptococcus sanguinis* (*S. sanguinis*) are instrumental in the initiation and progression of dental caries [5–7]. These bacteria facilitate caries development through mechanisms such as acidogenesis, biofilm formation, and adherence to dental surfaces. Furthermore, *Staphylococcus aureus* (*S. aureus*) has increasingly attracted attention as an opportunistic oral pathogen that colonizes the oral cavity and contributes to both localized and systemic infections [8–10]. The global economic impact of oral diseases is substantial, with annual expenditures estimated at ~710 billion USD, underscoring the urgent need for effective preventive and therapeutic interventions [11, 12].

Chlorhexidine is widely acknowledged as the gold standard for chemical plaque management due to its broad‐spectrum antimicrobial efficacy and substantivity [13]. Nevertheless, prolonged utilization of chlorhexidine is associated with various adverse effects, including dental discoloration, altered taste perception, mucosal irritation, and the potential emergence of microbial resistance [14–16]. These constraints have incited a heightened interest in natural products as alternative antimicrobial agents.

Plant extracts have garnered attention for their diverse array of bioactive compounds and relatively low toxicity profiles [17–19]. Numerous in vitro investigations have indicated that plant‐derived extracts can inhibit the proliferation and biofilm formation of cariogenic bacteria, suggesting their viability as natural substitutes [20–22]. The antimicrobial efficacy of medicinal plants is frequently attributed to phytochemical constituents such as phenolic acids, flavonoids, and terpenoids, which compromise bacterial cell membranes and disrupt crucial metabolic pathways [23–25].

**Figure 1:**
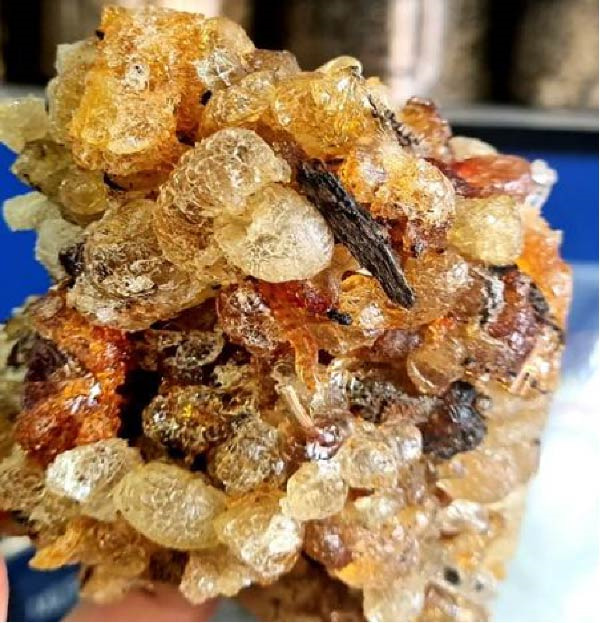
The natural gum exudate (Zedo gum) harvested from the Zagros Mountains in Kohgiluyeh and Boyer‐Ahmad Province, Iran.

*A. scoparia* is a shrub prevalent in the Zagros Mountains of Iran (Figure 1), characterized by the production of a natural gum abundant in polysaccharides and bioactive constituents [26]. Prior research has documented the antibacterial properties of extracts from this species against various microorganisms, including oral anaerobes [13, 17, 27]. However, the efficacy of its gum against predominant oral pathogens remains to be thoroughly explored. Consequently, the current investigation sought to assess the in vitro antibacterial activity of the hydroalcoholic extract derived from *A. scoparia* gum against *S. mutans*, *S. sobrinus*, *S. sanguinis*, and *S. aureus*, while comparing its effectiveness to chlorhexidine.

This study is significant because it evaluates the antibacterial activity of *A. scoparia* gum extract against key oral pathogens using both clinical and reference strains and compares its efficacy with chlorhexidine and conventional antibiotics. The findings may contribute to the development of natural antimicrobial agents for oral healthcare applications.

## 2. Material and Methods

### 2.1. Study Design

This study was approved by the Ethics Committee of Yasuj University of Medical Sciences, Yasuj, Iran (Ethics Code: IR.YUMS.BLC.1404.003) and was conducted in accordance with institutional ethical guidelines. Bacterial isolates used in this in vitro investigation were recovered from oral specimens without the collection of patient‐identifiable data. This experimental laboratory study was designed to evaluate the antibacterial effects of the hydroalcoholic extract of *A. scoparia* (Zedo) or Persian gum compared to chlorhexidine against oral pathogenic bacteria.

### 2.2. Study Population and Experimental Framework

The study included both clinical and standard reference strains of *S. mutans*, *S. sobrinus*, *S. sanguinis*, and *S. aureus*. Clinical isolates were harvested from oral biological samples (dental plaque and saliva), while reference strains (*S. mutans* ATCC 1683, *S. sanguinis* ATCC 1449, *S. sobrinus* ATCC 10556, and *S. aureus* ATCC 1112) were obtained from the Iranian Biological Resource Center (Tehran, Iran). Each bacterial isolate was subjected to treatment with the hydroalcoholic *A. scoparia* (Zedo) gum extract and 0.2% chlorhexidine. Antibacterial activity was quantified using three laboratory parameters: zone of inhibition (ZOI), minimum inhibitory concentration (MIC), and minimum bactericidal concentration (MBC). Each assay was performed in triplicate. The experimental design comprised a total of 162 trials, calculated as follows: 3 intervention groups × 6 bacterial isolate categories × 3 antibacterial indices × 3 replicates.

All procedures, including the processing of clinical isolates, preparation of the hydroalcoholic extract, bacterial cultivation, and susceptibility testing (well diffusion and MIC/MBC determination), were performed under standardized conditions in the Microbiology Laboratory of Yasuj University of Medical Sciences.

### 2.3. Plant Material Collection and Authentication

Gum from *A. scoparia* Spach (syn. *Prunus scoparia* Spach, Rosaceae) was collected on September 11, 2025 from Balout Bengan, a publicly accessible rangeland near Souq and Dehdasht, Kohgiluyeh and Boyer‐Ahmad Province, Iran (30.80°N, 50.39°E; 977 m altitude). The gum was harvested nondestructively from wild almond trees by Seyedeh Zahra Miri. Botanical identification was performed by Dr. Azizollah Jafari Koukhodan, a botanist at the Department of Botany, Yasuj University, Iran. A voucher specimen (Herbarium Number: Hyu1325‐632578) was deposited at the Yasuj University Herbarium for future reference. No specific permits were required for this academic collection from public land in accordance with national environmental regulations.

### 2.4. Preparation of *A. scoparia* (Zedo) Gum Extract

High‐purity, contaminant‐free Persian gum (Zedo) was harvested from the Kohgiluyeh region. The samples were thoroughly washed and air‐dried at ambient temperature, protected from direct sunlight, for several days. The desiccated gum was subsequently pulverized using a mortar and pestle. For the extraction process, 100 g of the gum powder was macerated in 1000 mL of a 70% ethanol–water solvent for 48 h at 37°C. The resulting solution was filtered through Whatman No. 1 filter paper and concentrated under reduced pressure using a rotary evaporator. The extract was then dried in an incubator at 45°C and stored at −20°C for subsequent experimental use [28, 29].

### 2.5. Preparation of Pathogenic Bacteria

Standard reference strains, including *S. mutans*, *S. sanguinis*, *S. sobrinus*, and *S. aureus*, were obtained as cryopreserved stocks in Tryptic Soy Broth (TSB) from the Iranian Biological Resource Center (Tehran, Iran). For reactivation, Streptococcus species were cultured on blood agar, while *S. aureus* was cultivated on Mueller–Hinton agar (MHA), followed by incubation at 37°C for 24 h. The identity of individual colonies was validated through standard biochemical assays, and cultures were preserved in TSB supplemented with 15%–20% glycerol at −70°C [30].

### 2.6. Identification Tests for Clinical Isolates

Clinical isolates were characterized using the following diagnostic criteria:

*S. aureus*: gram staining, catalase activity, mannitol fermentation on mannitol salt agar (MSA), and DNase production.

*S. mutans*: hemolytic patterns, optochin sensitivity, Voges‐Proskauer (VP) test, and urease activity.

### 2.7. Preparation of Microbial Suspension (0.5 McFarland Standard)

Bacterial suspensions were standardized to a 0.5 McFarland turbidity (~1.5 × 10^8^ CFU/mL). This was achieved by inoculating 3–4 pure, 24‐h‐old colonies into sterile physiological saline. The turbidity was monitored using a spectrophotometer at 625 nm, ensuring an optical density (OD) range of 0.08–0.13 [31].

### 2.8. Antibacterial Activity Assessment (Well Diffusion Assay)

The antibacterial efficacy of the Zedo extract and chlorhexidine was evaluated by measuring the ZOI. Standardized bacterial suspensions were spread onto MHA (for *S. aureus*) or blood‐supplemented MHA (for *Streptococcus* spp.) using sterile swabs. Wells (5 mm in diameter) were created using a sterile cork borer (5 mm) and filled with 20 µL of the Zedo extract or chlorhexidine at various concentrations. The extract solvent served as the negative control, while vancomycin (10 µg) and penicillin (10 µg) were utilized as positive controls. After incubation at 37°C for 18–24 h, the ZOI diameters were measured in millimeters. All assays were performed in triplicate to calculate the mean values.

### 2.9. Determination of MIC and MBC

The MIC and MBC were determined using the broth microdilution method in 96‐well microplates. Each well was filled with 100 µL of Mueller–Hinton Broth (MHB), followed by 100 µL of serially diluted Zedo extract or chlorhexidine. Subsequently, 10 µL of the standardized bacterial suspension was added to each well. Negative controls consisted of broth with either the extract or chlorhexidine (in the absence of bacteria) and broth alone. Positive controls included vancomycin and penicillin. The plates were incubated at 37°C for 24 h, and the MIC was identified as the lowest concentration preventing visible bacterial growth (turbidity). To determine the MBC, aliquots from wells showing no visible growth were subcultured onto MHA plates. The lowest concentration that prevented colony formation after 24 h of incubation was recorded as the MBC [32].

### 2.10. Statistical Analysis

Data were analyzed using SPSS software (Version 2024). Data normality was evaluated via the Kolmogorov–Smirnov test. For normally distributed data, parametric tests (independent *t*‐test or one‐way ANOVA) were employed to compare means. Nonparametric equivalents were used where the data deviated from normality. Statistical significance was predefined at *p* < 0.05.

## 3. Results

**Figure 2:**
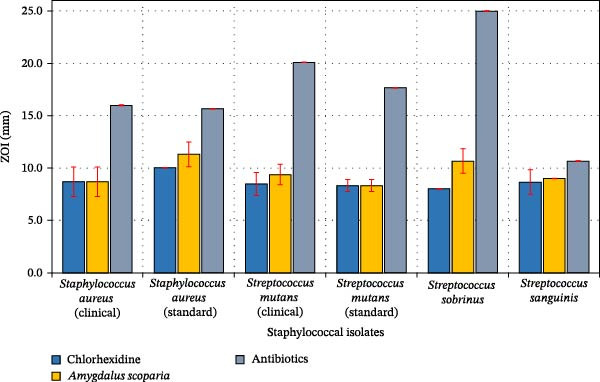
ZOI produced by 0.2% chlorhexidine, *A. scoparia* hydroalcoholic gum extract, and antibiotics (systemic positive controls) against clinical and standard isolates (*N* = 3).

**Figure 3:**
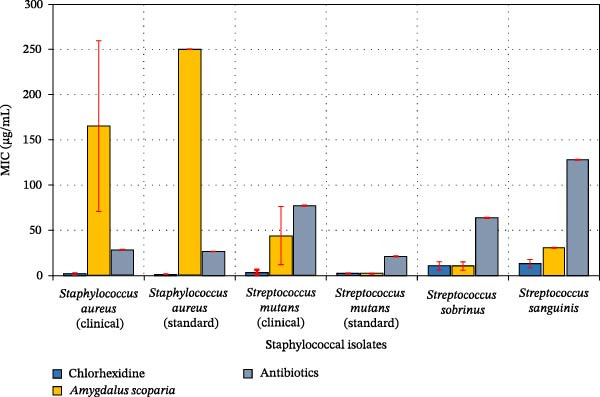
MIC of chlorhexidine, *A. scoparia* extract, and antibiotics against tested strains (*N* = 3).

**Figure 4:**
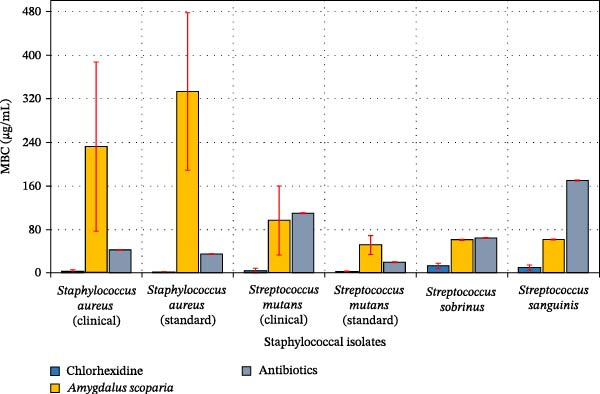
MBC of chlorhexidine, *A. scoparia* extract, and antibiotics against tested strains (*N* = 3).

The antibacterial efficacy of chlorhexidine, the hydroalcoholic gum extract of *A. scoparia*, and standard antibiotics (vancomycin and penicillin) was assessed against both clinical and standard isolates of *S. aureus*, *S. mutans*, *S. sobrinus*, *and S. sanguinis*. Efficacy was quantified through ZOI (Figure 2), MIC (Figure 3), and MBC (Figure 4) assays, with results presented as mean ± standard deviation (SD) based on triplicate experiments (Tables 1 and 2).

**Table 1:** Antibacterial activity of 0.2% chlorhexidine against clinical and standard isolates of oral bacterial strains (*n* = 3).

Bacterial species	Isolate type	ZOI (mm)	ZOI *p*‐value	MIC (µg/mL)	MIC *p*‐value	MBC (µg/mL)	MBC *p*‐value
*S. aureus*	Clinical	8.70 ± 1.42	0.7867^a^, <0.0001^b^	2.10 ± 1.10	0.001^a^, <0.0001^b^	3.60 ± 2.46	0.001^a^, 0.0028^b^
	Standard	10.00 ± 0.00	0.3131^a^, 0.0247^b^	1.33 ± 0.58	<0.0001^a^, 0.0708^b^	1.67 ± 0.58	0.1030^a^, <0.0001^b^
*S. mutans*	Clinical	8.50 ± 1.08	0.0448^a^, <0.0001^b^	3.51 ± 2.70	0.0062^a^, 0.0979^b^	5.03 ± 4.61	0.0027^a^, 0.1523^b^
	Standard	8.33 ± 0.58	0.9999^a^, 0.0014^b^	2.00 ± 0.00	0.0734^a^, 0.1220^b^	3.33 ± 1.15	>0.05^a^, >0.05^b^
*S. sobrinus*	Standard	8.00 ± 0.00	>0.05^a^, 0.05^b^	10.67 ± 4.62	0.0047^a^, 0.0043^b^	13.33 ± 4.62	0.0053^a^, 0.0049^b^
*S. sanguinis*	Standard	8.67 ± 1.15	>0.05^a^, >0.05^b^	13.33 ± 4.62	0.04^a^, <0.0001^b^	10.67 ± 4.62	0.0047^a^, 0.1216^b^

^a^ *p*‐Value compared to *A. scoparia* extract.

^b^ *p*‐Value compared to antibiotics (vancomycin + penicillin as systemic positive controls).

**Table 2:** Activity of *A. scoparia* gum hydroalcoholic extract against clinical and standard isolates of oral bacterial strains (*n* = 3).

Bacterial species	Isolate type	ZOI (mm)	ZOI *p*‐value	MIC (µg/mL)	MIC *p*‐value	MBC (µg/mL)	MBC *p*‐value
*S. aureus*	Clinical	8.70 ± 1.42	0.7867^a^, <0.0001^b^	165.00 ± 94.24	0.001^a^, <0.0001^b^	232.25 ± 155.32	0.001^a^, 0.0028^b^
	Standard	11.33 ± 1.15	0.3131^a^, 0.0414^b^	250.00 ± 0.00	<0.0001^a^, 0.0708^b^	333.33 ± 144.34	0.1030^a^, <0.0001^b^
*S. mutans*	Clinical	9.40 ± 0.97	0.0448^a^, <0.0001^b^	44.55 ± 31.97	0.0062^a^, 0.0979^b^	96.90 ± 63.25	0.0027^a^, 0.1523^b^
	Standard	8.33 ± 0.58	0.9999^a^, 0.0092^b^	2.00 ± 0.00	0.0734^a^, 0.1220^b^	52.00 ± 17.32	>0.05^a^, >0.05^b^
*S. sobrinus*	Standard	10.67 ± 1.15	>0.05^a^, >0.05^b^	10.67 ± 4.62	0.0047^a^, 0.0043^b^	62.00 ± 0.00	0.0053^a^, 0.0049^b^
*S. sanguinis*	Standard	9.00 ± 0.00	>0.05^a^, >0.05^b^	31.00 ± 0.00	0.04^a^, <0.0001^b^	62.00 ± 0.00	0.0047^a^, 0.1216^b^

^a^ *p*‐Value compared to chlorhexidine.

^b^ *p*‐Value compared to antibiotics (vancomycin and penicillin as systemic positive controls).

### 3.1. Antibacterial Effects on *S. aureus*

The antibacterial activity of 0.2% chlorhexidine and *A. scoparia* gum hydroalcoholic extract was evaluated against clinical and standard isolates of *S. aureus* (Tables 1 and 2). In terms of the ZOI (Figure 2), the *A. scoparia* extract and chlorhexidine demonstrated comparable effects against the clinical *S. aureus* isolate, both yielding a mean ZOI of 8.70 ± 1.42 mm with no significant difference between them (*p* = 0.7867). For the standard isolate, the extract produced a slightly larger ZOI (11.33 ± 1.15 mm) compared to chlorhexidine (10.00 ± 0.00 mm); however, this difference was not statistically significant (*p* = 0.3131). Despite the similar ZOI outcomes, the MIC (Figure 3) and MBC (Figure 4) assessments revealed a marked disparity in the actual concentrations required for antibacterial action. Chlorhexidine exhibited strong efficacy, requiring significantly lower concentrations to inhibit and kill the clinical isolate (MIC: 2.10 ± 1.10 µg/mL; MBC: 3.60 ± 2.46 µg/mL) compared to the *A. scoparia* extract (MIC: 165.00 ± 94.24 µg/mL; MBC: 232.25 ± 155.32 µg/mL), with statistically significant differences observed for both MIC and MBC (*p* = 0.001). A consistent trend was noted for the standard isolate, where chlorhexidine required substantially lower concentrations for inhibition (MIC: 1.33 ± 0.58 µg/mL) and eradication (MBC: 1.67 ± 0.58 µg/mL) than the extract (MIC: 250.00 ± 0.00 µg/mL and MBC: 333.33 ± 144.34 µg/mL). The difference in MIC between the two agents against the standard isolate was highly significant (*p* < 0.0001), whereas the difference in MBC was not statistically significant (*p* = 0.1030). Furthermore, when compared to the systemic positive controls (vancomycin and penicillin), both chlorhexidine and the *A.scoparia* extract showed significant differences in MIC and MBC against the clinical isolate (*p* < 0.0001 and *p* = 0.0028, respectively). For the standard isolate, the MIC values of both test agents did not differ significantly from the antibiotics (*p* = 0.0708), but their MBC values were significantly different (*p* < 0.0001). Overall, while both agents displayed similar ZOIs, chlorhexidine possessed a considerably higher intrinsic antibacterial potency against *S. aureus* than the *A. scoparia* extract.

### 3.2. Antibacterial Effects on *S. mutans*

Against *S. mutans*, the systemic antibiotics produced the most substantial ZOIs (Figure 2) for both clinical (20.80 ± 2.53 mm) and standard (17.67 ± 0.58 mm) isolates, which were significantly larger than those induced by 0.2% chlorhexidine and the *A. scoparia* extract. When comparing the two test agents, the *A. scoparia* extract yielded a significantly larger ZOI than chlorhexidine against the clinical isolate (9.40 ± 0.97 mm vs. 8.50 ± 1.08 mm; *p* = 0.0448), whereas no significant difference was observed for the standard strain (both 8.33 ± 0.58 mm; *p* = 0.9999). Conversely, in terms of MIC (Figure 3), chlorhexidine exhibited markedly superior potency, requiring significantly lower concentrations to inhibit the clinical isolate compared to the extract (3.51 ± 2.70 µg/mL vs. 44.55 ± 31.97 µg/mL; *p* = 0.0062), although the difference was not statistically significant for the standard strain (2.00 ± 0.00 µg/mL for both; *p* = 0.0734). In comparison, the antibiotic controls required higher MICs (77.60 ± 100.21 µg/mL for clinical and 21.33 ± 9.24 µg/mL for standard isolates). A consistent trend was observed for bactericidal activity (MBC, Figure 4), where chlorhexidine was significantly more effective than the extract against the clinical isolate (5.03 ± 4.61 µg/mL vs. 96.90 ± 63.25 µg/mL; *p* = 0.0027), with no significant difference noted for the standard strain (3.33 ± 1.15 µg/mL vs. 52.00 ± 17.32 µg/mL; *p* > 0.05). An interesting discrepancy emerged between the diffusion and dilution assays; despite requiring substantially higher concentrations to inhibit bacterial growth, the *A. scoparia* extract produced a significantly larger ZOI than chlorhexidine against the clinical *S. mutans* isolate (Figure 2, Tables 1 and 2).

### 3.3. Antibacterial Effects on *S. sobrinus* and *S. sanguinis*

Against the standard *S. sobrinus* strain, the systemic antibiotics produced the largest ZOI (25.00 ± 5.00 mm), which was marginally larger than that of 0.2% chlorhexidine (8.00 ± 0.00 mm; *p* = 0.05) but not significantly different from the *A. scoparia* extract (10.67 ± 1.15 mm; *p* > 0.05) (Tables 1, 2 and Figure 2). Interestingly, both chlorhexidine and the *A. scoparia* extract exhibited identical mean MICs (10.67 ± 4.62 µg/mL for both) (Tables 1, 2 and Figure 3). However, chlorhexidine demonstrated a significantly stronger bactericidal effect, requiring a substantially lower MBC (13.33 ± 4.62 µg/mL) compared to the extract (MBC: 62.00 ± 0.00 µg/mL; *p* = 0.0053).

For the standard *S. sanguinis* isolate, no statistically significant differences in ZOI were observed among chlorhexidine (8.67 ± 1.15 mm), the *A. scoparia* extract (9.00 ± 0.00 mm), and the antibiotic controls (10.67 ± 1.15 mm; all *p* > 0.05). However, in terms of inhibitory activity, chlorhexidine required a significantly lower concentration (MIC: 13.33 ± 4.62 µg/mL) compared to the *A. scoparia* extract (MIC: 31.00 ± 0.00 µg/mL; *p* = 0.04). Furthermore, chlorhexidine exhibited a significantly stronger bactericidal effect (MBC: 10.67 ± 4.62 µg/mL) than the extract (MBC: 62.00 ± 0.00 µg/mL; *p* = 0.0047) (Tables 1, 2 and Figure 4).

### 3.4. Resistance Patterns in Clinical vs. Reference Strains

A consistent observation across all treatments was that clinical isolates *of S. aureus and S. mutans* demonstrated greater resistance than their standard counterparts, characterized by higher MIC/MBC values and smaller ZOI (Figure 2) measurements. While certain MIC and MBC data showed zero SD (indicating highly consistent responses in *S. sobrinus* and *S. sanguinis*), the high SD values observed in clinical isolates reflect significant variability in antibacterial susceptibility (Figures 3 and 4). Overall, Figure 4 confirms that chlorhexidine consistently exhibited the lowest MBC values across all tested isolates, affirming its potent bactericidal activity.

## 4. Discussion

Growing concerns regarding the adverse effects of chlorhexidine—including dental staining, alterations in taste perception, and the potential emergence of microbial resistance—have intensified interest in natural antimicrobial alternatives for oral healthcare [14–16]. A pivotal finding of the current study is that the hydroalcoholic extract of *A. scoparia* gum produced a significantly larger inhibition zone against clinical isolates of *S. mutans* compared to 0.2% chlorhexidine (9.40 ± 0.97 mm vs. 8.50 ± 1.08 mm; *p* = 0.0448).

This observation is of substantial clinical relevance, as *S. mutans* serves as a primary etiological agent in the pathogenesis of dental caries. Furthermore, clinical isolates of this pathogen frequently exhibit enhanced phenotypic resistance compared to standardized laboratory strains. Phytochemical characterization via gas chromatography–mass spectrometry (GC–MS) identified several bioactive compounds within the extract, notably phenolic acids (e.g., gallic acid), flavonoids (e.g., quercetin), and terpenoids (e.g., β‐pinene). These results align with established phytochemical profiles for the *Prunus/Amygdalus* genus reported in prior literature [21–23].

### 4.1. Mechanisms of Action

These bioactive constituents are recognized for their multitargeted antimicrobial efficacy. Their mechanisms of action include the disruption of bacterial membrane integrity, attenuation of acidogenesis, interference with essential enzymatic processes, and the suppression of extracellular polysaccharide (EPS) biosynthesis, which is fundamental to biofilm architecture [15, 16].

### 4.2. Comparative Antibacterial Efficacy

While chlorhexidine demonstrated significantly greater potency, with MIC and MBC values in the microgram‐per‐milliliter range, the plant extract required milligram‐per‐milliliter concentrations to achieve equivalent inhibitory outcomes. This discrepancy is typical for crude botanical extracts, which comprise complex assemblies of phytochemicals where individual active agents are present at relatively low titers [13, 21, 22]. These results are consistent with Talei et al. [13], who observed MICs between 25 and 40 mg/mL for *A. scoparia* extracts against oral anaerobes [10]. Furthermore, Mehdipour et al. [22] and Zare et al. [21] reported that several Iranian medicinal plants exhibit moderate antibacterial activity when benchmarked against chlorhexidine. The current findings corroborate these previous studies, reinforcing the viability of indigenous medicinal plants as prospective reservoirs for alternative antimicrobial therapeutics in dentistry.

### 4.3. Diffusion Assays vs. Dilution Methods

Notably, plant extracts may yield ZOIs comparable to or exceeding those of chlorhexidine, despite possessing higher MIC or MBC values [20]. Diffusion‐based methodologies, such as the agar well diffusion method, may more accurately represent the localized efficacy of complex phytochemical mixtures. In these assays, the physical diffusion characteristics and potential synergistic interactions between various constituents can amplify the observed inhibitory area. The substantial ZOI recorded against clinical *S. mutans* isolates in this study supports the hypothesis that diffusion‐based metrics capture unique aspects of extract performance. A critical factor contributing to this discrepancy lies in the distinct physicochemical properties of the test agents. Chlorhexidine is a polycationic (positively charged) bisbiguanide molecule that tends to bind electrostatically to negatively charged components within the agar matrix (such as sulfate groups in agarose). This strong interaction significantly restricts the lateral diffusion rate of Chlorhexidine through the agar, thereby limiting the physical size of its inhibition zone despite its high intrinsic potency. Conversely, the bioactive low‐molecular‐weight phytochemicals in the *A. scoparia* extract, such as phenolic acids and flavonoids, possess different diffusion kinetics and can migrate more freely through the medium. Furthermore, the complex mixture of phytochemicals in the extract may exhibit synergistic interactions as they diffuse, creating a combined inhibitory gradient that amplifies the observed ZOI. Thus, the larger ZOI reflects favorable diffusion characteristics and localized synergistic effects rather than superior absolute bactericidal potency.

### 4.4. Clinical Significance of *S. aureus*

Although *S. aureus* is not classified as a primary oral pathogen, its role as an opportunistic agent in peri‐implantitis, advanced periodontitis, oral mucosal pathologies, and infections within immunocompromised populations is increasingly recognized [33, 34]. Consequently, incorporating this bacterium into the current experimental design was clinically warranted, serving as a robust Gram‐positive model to evaluate the broader antibacterial spectrum of the *A. scoparia* extract.

### 4.5. Clinical Isolates vs. Reference Strains

A significant finding was the consistently higher resistance profile exhibited by clinical isolates relative to standard reference strains. This trend, well‐documented across antimicrobial research, underscores the necessity of utilizing clinical isolates in laboratory frameworks to more accurately simulate real‐world microbial dynamics and therapeutic challenges [35–37].

### 4.6. Study Limitations and Future Perspectives

Despite these encouraging outcomes, several limitations must be acknowledged. Primarily, the strictly in vitro nature of this investigation does not fully encapsulate the intricate biological milieu of the human oral cavity. Additionally, the study did not account for biofilm dynamics or the influence of salivary interactions, both of which are critical to the oral microbial ecology. The cytotoxicity of the extract on oral tissues also remains uncharacterized, and the quantitative phytochemical profiling was nonexhaustive. Future investigations should prioritize advanced biofilm modeling, in vivo experimental designs, standardized phytochemical quantification via GC–MS, and subsequent clinical trials to comprehensively evaluate the therapeutic utility of *A. scoparia* gum.

## 5. Conclusion

In summary, the hydroalcoholic gum extract of *A. scoparia* exhibited substantial in vitro antibacterial efficacy against primary cariogenic streptococci and the opportunistic pathogen *S. aureus*. Notably, the extract yielded a statistically larger ZOI against clinical isolates of *S. mutans* compared to the 0.2% chlorhexidine control (*p* = 0.0448).

While the MIC and MBC values for the extract were higher than those of chlorhexidine, its rich phytochemical composition and superior performance in diffusion‐based assays underscore its potential for integration into preventive oral healthcare formulations.

However, since this investigation was conducted under in vitro conditions with a limited number of isolates and lacked toxicological or clinical assessments, further research is warranted. Future studies focusing on in vivo validation, formulation development, safety profiling, and the elucidation of molecular mechanisms are essential to confirm the definitive therapeutic utility of this botanical extract.

## Author Contributions

Seyedeh Zahra Miri contributed to the investigation, data curation, methodology, and writing – review and editing. Fatemeh Khajehzadeh contributed to the conceptualization, supervision, project administration, methodology, writing – original draft, correspondence, and had full access to all the data in the study and takes full responsibility for the integrity of the data and the accuracy of the data analysis. Arash Asfaram contributed to the methodology, formal analysis, visualization, resources, investigation (extraction procedures), data analysis, and writing – review and editing. Razieh Dehbanipour contributed to the investigation, resources, laboratory assistance, validation, methodology, and writing – review and editing. Mobina Moeindoost contributed to the investigation, resources, laboratory assistance, and writing – review and editing.

## Funding

This research did not receive any specific grant from funding agencies in the public, commercial, or not‐for‐profit sectors.

## Disclosure

All authors have read and approved the final version of the manuscript. The study was conducted as part of a postgraduate thesis at the Yasuj University of Medical Sciences.

## Ethics Statement

This study was approved by the Ethics Committee of Yasuj University of Medical Sciences, Yasuj, Iran (Ethics Code IR.YUMS.BLC.1404.003). The study was conducted in accordance with institutional ethical guidelines. Bacterial isolates used in this in vitro investigation were obtained from oral samples without recording any patient‐identifiable information.

## Conflicts of Interest

The authors declare no conflicts of interest.

## Data Availability

The authors confirm that the data supporting the findings of this study are available within the article.
